# Dead and Diseased Teeth and Their Treatment

**Published:** 1888-10

**Authors:** E. S. Niles


ARTICLE VI.

### DEAD AND DISEASED TEETH AND THEIR TREATMENT.

DR. E. S. NILES.

For convenience, I shall divide my subject into four classes :—

1. Teeth with central nerves and vessels extirpated, and free from internal and external septic infiltration with the peridental membrane performing its normal function.

2. Teeth with their central nerves and vessels destroyed, and their decomposed remains unremoved, and septic infiltrations extending to the peridental membrane, which becomes inflamed at the slighest depressing influence on the general system.

3. The death of the central pulp, followed by putrefaction and infiltration of all the dentinal tissue, the cemental tissues infected, inflamed, thickened, and a connective tissue wall formed protecting deeper parts of the alveolus. Tnis wall is usually located at the foramina at the apex of the root, and is generally known as the "secreting sac," leading from which is the fistulous passage opening to the surface offering the least resistance of tissue. When this condition is allowed to continue for years, the root membrane is destroyed, and even the root itself is absorbed wholly or in part.

A tooth becomes dead when it loses its membrane as well as its central pulp. These teeth I have designated as the 4th class. Their destruction is rarely due to the death of the pulp by internal causes, but more especially from outward wasting of tissues, from calculus or diseased alveolar tissues, when without support such teeth may be called dead irritating, foreign bodies.

In the four classes of diseased teeth mentioned we observe variable conditions, any one of which of the milder forms may be but the progressive stages of the same trouble which if not arrested will lead to the loss of that tooth, if not to more grave results. A tooth if deprived of its central nerve and blood supply resists destructive agencies of decay by its chemical integrity, in so far as this integrity is not overcome by what may be called "greater chemical affinities." As it becomes septic, within the pulp-chamber, its destructive tendencies are two-fold; it is "a house divided against itself," and the only agents necessary to support and carry on the destruction of the tissues are water and air. The products of this change, as we find them, are deprived primarily from the pulp matter, but later from the breaking down of the organic substance of the tubuli. The main portion of the pus, however, in the progressive stages, is derived from the diseased or infected tissues immediately at the apex of the root, the fistulous opening usually appearing on the surface of the gum, carrying or furnishing vent to the matter and gas to which it auguments. The generation of gases accompanying the breaking down of the dental tissues is, for the most part, the same as those generated from decomposing organic bodies whose constituents are largely of phosphorus matter, as the brain, bone or fish. This gas I apprehend to be phosphoretted hydrogen. It is found also in closed cavities of decaying teeth, and is significant of decomposing organic and inorganic tooth structure into ultimate proximate principles, as lime, magnesium, phosphoric acid, or phosphorus, and probably the re- formation of other substances, as in the case of phosphoretted hydrogen.

If we are to weigh the questions of life or the death of an organ, our attention is first called to known arrangements of tissues, that involved in death and that living, and we draw our conclusions as to the vitality on which rests our chief reliance for support; we look at the condition of a tooth without central blood-vessel or nerve supply, and we see at once the only reliance for vital support is from the cemental tissues, and all treatment must be directed to preserve these tissues from the destructive processes progressing in the dentine. When once this infectious matter has invaded the pulp chamber and tubuli, the tooth becomes ever after an easy prey to these changes, and in no way can it be kept so free from septic influences as to prevent it from becoming septic.

For various reasons teeth do die, and we find dead pulps resulting from numerous causes, and though not always septic, they are liable to become so if at all exposed. It is well known that a devitalized pulp may not cause any irritation, either during death or for years after, but this cannot be regarded as evidence that such teeth should not be disinfected, cleaned and sealed up, but should be regarded rather as an indication of the power of the geneaal health to take up or absorb dead matter. Such power, however, may or may not be present at different periods of life. For instance, a tooth may remain quiet for years, and then suddenly enter upon acute inflammation and terminate in a chronic cold abscess. The poisonous matter generated from the dentine having infected or poisoned the soft tissues, the system no longer able to carry on the absorbing process, it expels the matter and provides for future accumulation by a fistulous opening and a connective tissue wall around the infected parts.

We have thus described the second class of teeth named, and indicated the progressive stages of septic influences which render the tooth more and more irritating to the surrounding parts. We often find that the fistula, if probed, leads to a large cavity immediately at the end of the root, and the root, denuded of its membrane, furnishes a portion of the wall to the cavity named.

TREATMENT.—When the cause of the trouble and the conditions of tissue are well understood, the question of treatment is often simple. With the present difficulty and the means at hand, barring mal-formation of the roots, the chances of success are more than equal to any operation we are called upon to perform. As in all diseases of the system, if our patient is strong and vigorous, however long the abscess has existed, fifty per cent of success is assured. On the other hand, desired results are not so speedily obtained in more delicate or enfeebled health. In all cases, relief from pain is the first step in treatment; often an aching, exposed pulp may, by the use of narcotics, be treated into rest and the pulp destroyed.

For the destruction of pulps, I have found for the present, nothing that meets the wants of the case so well as arsenious acid, though I am of the opinion that a better preparation can be prepared. That used by me is composed of equal parts of arsenious acid and the acetate or sulphate of morphia, made into a paste with carbolic acid or creosote. Right here is danger. Many inflamed peridental membranes are caused primarily by arienic preparations getting through the apex o, the root and creating dead tissue and aseptic influences. It is better to cause the patient a little pain than to run the risk of a diseased membrane. I have used nitrate of silver with very favorable results. It does not cause pain, and its destruction of tissue is immediate.

With the usual means, the roots are cleared of all the pulp possible, dried with hot air till there is no moisture; not only the pulp canals, but the dentinal tubes should be dried as far in as possible, after which the alcohol may enter even to the inter-globular spaces. In filling, I make use of the well-known preparation of gutta-percha and chloroform. In the treatment of abscessed and septic teeth, of course a long cleansing process is necessary. I strongly oppose the method of opening through the apex of the root, as by so doing there is great liability of further infecting the tissues by means of the infected drill, though I use the drill to get a free opening as near the apex as possible. This done, the canals are washed thoroughly with warm water and dried with hot air, after which is saturated with a solution of strong bichloride of mercury (five per cent), the opening in the crown being stopt with gutta-percha. This is repeated once in three or four days till there is no smell of phosphoretted hydrogen, and I am satisfied that the tooth is thoroughly aseptic, when it is sealed and filled as in the previous case.

After a portion of the peridental membrane has been destroyed, is it possible to render the cemental structure so aseptic that the alveolar tissues will harden about the exposed surface without irritation? Of course there is not assurance of success as in the previous cases, but in the mouth of the average person of health, the bichloride treatment should be tried outside and in. I have been surprised at the results of the cases treated; out of three, two have been successful; the other was a case where I could hardly expect success; so extensive was the trouble that three of the superior centrals had their roots partially absorbed, and the only course seemed to be extraction, which, after the operation, I did not regret at all. Though badly diseased and absorbed, the alveolus healed very quickly without treatment.

It is hardly necessary for me to say that my treatment for the fourth class of teeth named is extraction, as they are indeed "dead teeth" and "foreign irritating bodies."—*Independent Practitioner*.

---

ARTICLE VII.

